# Hydrogel-Based 3D Bioprinting Technology for Articular Cartilage Regenerative Engineering

**DOI:** 10.3390/gels10070430

**Published:** 2024-06-28

**Authors:** Hongji Zhang, Zheyuan Zhou, Fengjie Zhang, Chao Wan

**Affiliations:** 1 Key Laboratory of Regenerative Medicine, Ministry of Education, School of Biomedical Sciences, Faculty of Medicine, The Chinese University of Hong Kong, Hong Kong SAR, China; zhan1754@link.cuhk.edu.hk (H.Z.); zheyuanzhou@cuhk.edu.hk (Z.Z.); b123228@cuhk.edu.hk (F.Z.); 2 Center for Neuromusculoskeletal Restorative Medicine, Hong Kong Science Park, Hong Kong SAR, China; 3 Key Laboratory of Regenerative Medicine (Shenzhen Base), Ministry of Education, School of Biomedical Sciences Core Laboratory, Institute of Stem Cell, Genomics and Translational Research, Shenzhen Research Institute, The Chinese University of Hong Kong, Shenzhen 518057, China

**Keywords:** three-dimensional bioprinting, hydrogel, cartilage, bioink, biomaterials, tissue engineering

## Abstract

Articular cartilage is an avascular tissue with very limited capacity of self-regeneration. Trauma or injury-related defects, inflammation, or aging in articular cartilage can induce progressive degenerative joint diseases such as osteoarthritis. There are significant clinical demands for the development of effective therapeutic approaches to promote articular cartilage repair or regeneration. The current treatment modalities used for the repair of cartilage lesions mainly include cell-based therapy, small molecules, surgical approaches, and tissue engineering. However, these approaches remain unsatisfactory. With the advent of three-dimensional (3D) bioprinting technology, tissue engineering provides an opportunity to repair articular cartilage defects or degeneration through the construction of organized, living structures composed of biomaterials, chondrogenic cells, and bioactive factors. The bioprinted cartilage-like structures can mimic native articular cartilage, as opposed to traditional approaches, by allowing excellent control of chondrogenic cell distribution and the modulation of biomechanical and biochemical properties with high precision. This review focuses on various hydrogels, including natural and synthetic hydrogels, and their current developments as bioinks in 3D bioprinting for cartilage tissue engineering. In addition, the challenges and prospects of these hydrogels in cartilage tissue engineering applications are also discussed.

## 1. Introduction

Osteoarthritis (OA) is one of the most common degenerative joint diseases with a high disability rate, whose incidence increases with an aging population and increasing obesity rates. OA is becoming increasingly prevalent. According to clinical statistics, about 250 million people worldwide are suffering from OA. Up to now, the detailed pathogenesis of OA is still not fully understood, and the efficacy of existing therapeutic strategies cannot meet the requirements of patients in clinics [1,2].

Natural cartilage tissue is avascular and nerve-free, which limits its ability to repair itself. It has been reported that articular cartilage defects over 2 cm^2^ in size in the human knee are hardly self-repairing [3]. Early treatments mainly include cartilage drilling/microfracture, autologous/allogeneic chondrocyte transplantation, and joint replacement [4,5]. However, these strategies have failed to meet clinical requirements owing to immunological rejection, insufficient donor sources, or poor long-term results. In order to avoid these defects, tissue engineering becomes an effective approach to improve cartilage repair or regeneration by loading cells in the form of scaffolds, greatly avoiding immune rejection [6,7]. However, as far as the structure and performance are concerned, the engineered cartilage is hardly the same as natural cartilage, which affects its function in cartilage repair. Using the traditional engineering approaches, the bio-mimic structure of articular cartilage is difficult to be rebuilt due to the fact that natural cartilage tissue has a complicated multilayered structure, with different cell morphology and complicated extracellular matrix (ECM) components [8]. In recent years, the rise and maturity of 3D bioprinting technology, have provided a new technology platform for tissue engineering and regenerative medicine. The 3D bioprinting technology is based on computer-aided bioprinters to fabricate bioscaffolds with cells or bioactive factors incorporated. It can precisely assemble biomaterials with cells and construct the required 3D structures according to the clinical needs [9,10].

**Figure 1:**
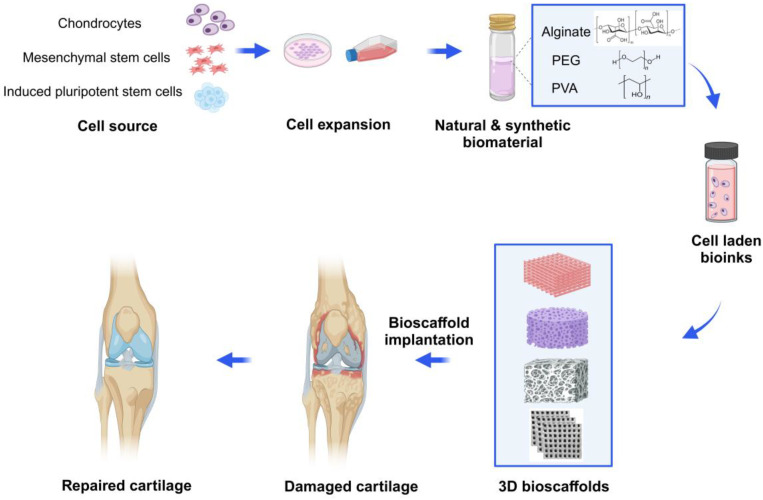
Schematic diagram of 3D bioprinted hydrogel for articular cartilage tissue engineering. Chondrocytes, mesenchymal stem cells (MSCs) or chondroprogenitor cells are isolated, cultured and expanded with the growth medium. Then, the cells are incorporated with natural hydrogels (e.g., alginate) or synthetic hydrogels (e.g., PEG or PVA) and create various 3D-printed bioscaffolds using the commonly used bioprinting strategies in tissue engineering. Then, according to specific circumstances and needs, the bioscaffolds are transplanted into the damaged cartilage, enabling articular cartilage to repair and regenerate. Created with BioRender.com.

Three-dimensional bioprinting relies on two main factors, including (1) the design of bioink as precursors (biomaterials, cells, growth factors and drugs); (2) the use of the 3D bioprinting method to build natural tissue mimicking models with various physicochemical features and tunable pore structures [11,12]. Despite the limitations of common methods, 3D bioprinting techniques can fabricate 3D bioscaffolds with high-precision, various morphology and structure, controlled interconnected porosities (in terms of size, shape, and volume), and designed material composition, along with different cells that can mimic the ECM properties. By using autologous cells to fabricate 3D-bioprinted tissues or organs, immune rejection can be significantly avoided, and the ethical issues are greatly reduced [13,14]. At present, the 3D bioprinting technology has been widely used in cartilage repair or regeneration. Furthermore, more research is dedicated to the functional mimicry of engineered cartilage tissue, thereby accelerating the process of its clinical translation [15,16] (Figure 1).

In this review, we mainly summarized the current progress of different types of hydrogel biomaterials, including natural and synthetic hydrogels used for articular cartilage repair or regeneration. The challenges and improvement directions of these hydrogels in cartilage tissue engineering applications are also stated.

## 2. Traditional Approaches for Articular Cartilage Repair or Regeneration

**Figure 2:**
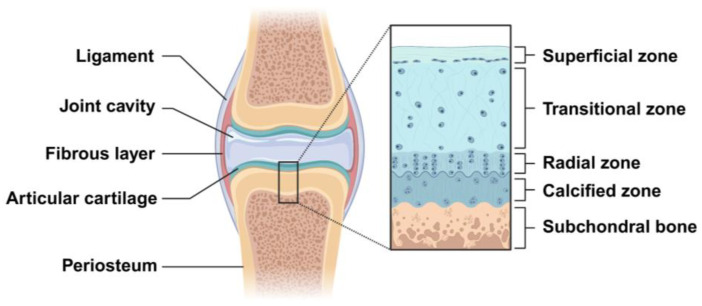
Schematic representation of articular cartilage structures in the knee joint. The structure of articular cartilage is organized into four distinct zones: the superficial, transitional, radial, and calcified zones. The calcified cartilage lies between the subchondral bone and the radial zone. Created with BioRender.com.

Cartilage is a resilient, avascular and aneural connective tissue, consisting of chondrocytes in an ECM that is rich in collagen type II and proteoglycan. Some studies indicate that the average thickness of human adult articular cartilage (hyaline articular cartilage layer) is about 2.4 mm and is significantly different with age. Below 40 years (16–40 years) of age, the full thickness of the articular cartilage ranges from 2.01 to 2.89 mm, with an average of 2.34 ± 0.30 mm, while above 40 years (41–86 years) of age, the full thickness of the articular cartilage ranges from 1.57 to 2.79 mm, with an average of 2.23 ± 0.36 mm [17,18]. Articular cartilage can be histologically composed of four zones, which are named the superficial zone, transitional zone, radial zone, and calcified zone. The superficial zone is the thinnest layer of articular cartilage (10–20% of cartilage thickness) and is mainly composed of the flattened chondrocytes and three important protein molecules (lubricin, proteoglycan, hyaluronic acid), besides having a high water content. The transitional zone has more spherical chondrocytes and a relatively less water content, with proteoglycans and higher collagen fibrils. The radial zone is composed of many chondrocytes arranged in a columnar shape and has less water content, with larger proteoglycan and collagen fibrils [19]. The calcified cartilage is located between the articular cartilage and the subchondral bone (Figure 2). Therefore, the difference in the physical, mechanical, and biological properties between different zones is determined by the unique anatomical and histological structures of articular cartilage. Due to the avascular and aneural characteristics of cartilage, the regeneration of the cartilage lesions is very challenging.

In the fetal period, infancy, or childhood, articular cartilage has its own regenerative activity. However, articular cartilage cannot be regenerated in the adults because mature chondrocytes do not proliferate and are long-lived within the body. If injuries occur in the subchondral bone, regeneration will be possible due to the proliferation of mesenchymal progenitor cells, located in the zone of the blood capillaries of bone tissue, and these cells can be differentiated into chondrocytes. In such cases, because of the formation of a large amount of fibrous cartilage tissue, the mechanical properties of the regenerative cartilage are not comparable to those of the natural articular cartilage. In hyaline articular cartilage (HAC), there are chondroblasts or perichondrial cells, which are mesenchymal progenitor cells that can differentiate into chondrocytes in the ECM and induce endochondral ossification [20,21].

It is well known that collagens in the ECM, such as collagen type II, IX, X, and XI determine the tensile strength of articular cartilage. The amount of water in articular cartilage and the number of chemical bonds present in collagen molecules decrease with age. As a result, the articular cartilage of the elderly is prone to weakened elasticity and lacks effective resistance to external forces, including stretching, twisting, and compression loads [22]. In other words, articular cartilage becomes more vulnerable to damage and induces the progression of OA [23].

At present, the conservative treatments for articular cartilage injury mainly include anti-inflammatory, analgesic, and drug therapy, in addition to weight-bearing and physical therapy. However, many treatments only alleviate the clinical symptoms and do not repair cartilage damage nor prevent joint destruction [24]. Therefore, in cases of severe cartilage injury, surgical treatment, such as chondroplasty or arthroplasty is required [25]. Chondroplasty is a minimally invasive surgical procedure used to repair damaged cartilage and includes microfracture surgery, drilling, mosaicplasty, allograft transplantation, and osteochondral autotransplantation [26,27]. However, there remain many deficits such as graft cell death or chondrocyte hypertrophy, graft malnutrition, and fibrocartilage formation. If the destruction of articular cartilage deepens, joint replacement surgery is required [28,29,30]. However, joint replacement may significantly increase the financial burden on the patients [31,32]. Besides, the risks and complications, which include instability of the artificial joint, blood clots, infection, and nerve damage need to be faced [33,34]. The failure of the prosthesis is another difficult situation that may require a more complicated revision surgery. Another approach is endoarthroscopy, which can detect a lot of joint problems, such as a torn meniscus or patella, but it has limitations in restoring the native elements of the joint.

Therefore, in view of the problems mentioned above, it is necessary to develop and optimize better methods to repair articular cartilage lesions. More importantly, the regenerated cartilage tissue has the same function as the natural cartilage, and it can better integrate and grow with the surrounding host cartilage tissue.

## 3. Tissue Engineering for Treatment of Cartilage Damages

Cartilage tissue engineering is an interdisciplinary field that applies the principles of engineering and life sciences to develop biological substitutes that restore, maintain, or improve the structural function of damaged cartilage [35]. Cartilage tissue engineering is an integrative approach incorporating chondrogenic cells, biomaterials, and bioactive molecules. In general, cartilage tissue engineering includes six steps. (1) Seed cells: the cells commonly used for cartilage tissue engineering include chondrocytes, periosteum-derived progenitor cells, mesenchymal stem cells and pluripotent stem cells. (2) Cell expansion: *in vitro* expansion of a large number of chondrogenic cells to meet the needs of generating clinical-scale engineered cartilage tissue. (3) Bioscaffolds: bioscaffolds provide structural support, enhance chondrogenesis, and guide the regeneration of cartilage. The ideal bioscaffolds have the characteristics of biocompatibility, durability, low immunogenicity, etc. Hydrogel materials generating bioscaffolds can be classified as natural and synthetic polymers. Cells and growth factors or cytokines were incorporated with these biomaterials to create 3D bioscaffolds. (4) Bioreactor: bioreactors are the desirable device to mimic multiple characteristics of the *in vivo* milieu. It often controls the pH level, temperature, nutrient supply, oxygen level, waste removal and the use of mechanical, electrical or magnetic stimulation. Under exposure to bioreactors, the chondrogenitor cells or MSCs/pluripotent stem cells will differentiate into chondrocytes, which are capable of synthesizing and depositing collagen type II to guarantee tissue stability. Using a bioreactor for screening tissue-engineered cartilage constructs can accelerate the translational process for more clinically relevant research. (5) Pre-clinical test: bioscaffolds are incubated *in vitro* for several weeks and implanted in nude/SCID mice *in vivo* for long term study. (6) Cartilage repair or regeneration: engineered bioscaffolds are implanted at the site of damage and gradually integrate with the surrounding host tissue to restore cartilage function [36,37].

## 4. Three-Dimensional Bioprinting Materials for Articular Cartilage Repair or Regeneration

Up to now, 3D printing technology has been considered to be one of the most promising technologies for cartilage tissue engineering, replacing damaged or non-functional cartilage tissue with 3D-printed biomaterials and cells. A variety of 3D bioprinted porous bioscaffolds serve as cartilage ECM to promote nascent cartilage formation. The 3D porous bioscaffold is composed of three main components, including biomaterials, cells (chondrocytes or MSCs), and bioactive molecules such as growth factors, cytokines, or small molecule compounds. It provides an exoskeleton for cartilage formation and regeneration [38,39,40]. The ideal 3D printing process mainly includes short printing time, high printing resolution, and good biocompatibility of the materials. Histomorphologically, the bioprinted bioscaffolds should be able to induce cell differentiation and migration, thus affecting ECM deposition and greatly displaying properties that are similar to those of natural tissues [41]. In addition, 3D printing technology also allows the porosity, internal structure, mechanics, and structural characteristics of the bioprinted product to be finely adjusted by controlling their bioprinting processes [42].

**Figure 3:**
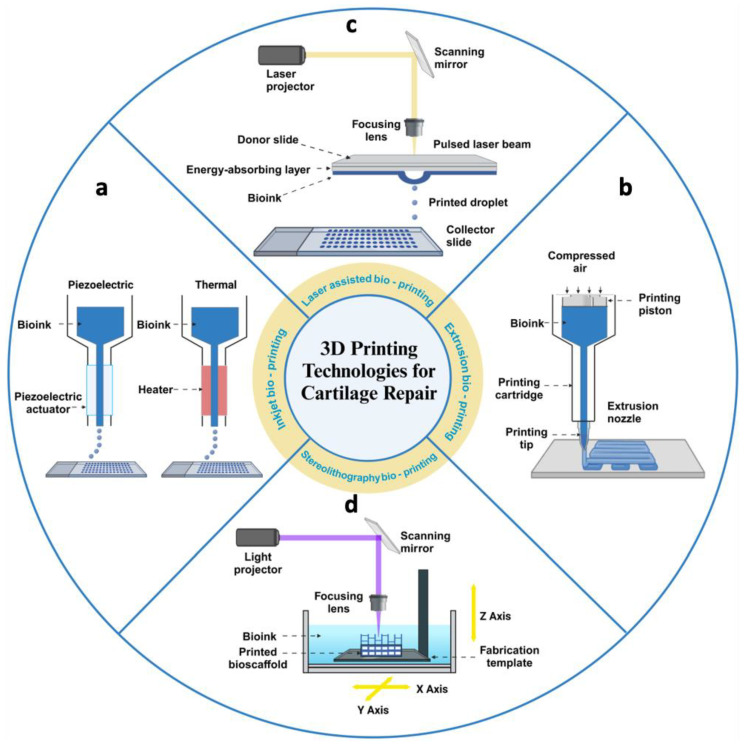
Schematic diagram of four methods of 3D bioprinting for articular cartilage tissue engineering. (**a**) Inkjet printers extrude cells and bioinks in the form of droplets, driven by thermal energy or piezoelectricity. (**b**) Extrusion printers deposit cells and bioinks in a continuous extrusion into 3D structures by pneumatic or mechanical screw actuation. (**c**) Laser-assisted printers use laser pulses to act on an energy-absorbing layer to create high-pressure bubbles that deposit droplets of bioinks with cells onto the substrate. (**d**) Stereolithographic printers use optical projection technology to cure bioinks with cells layer by layer. Created with BioRender.com.

Currently, a variety of technologies have been developed to fulfil the requirements of 3D bioprinting. Among them, the most commonly used methods are extrusion-based bioprinting, inkjet bioprinting, laser-based bioprinting, and stereolithography bioprinting [10,43] (Figure 3).

(1) Extrusion-based bioprinting methods typically use a pneumatic actuator or screw device to feed material from a cartridge through a nozzle or needle for deposition. These methods, compatible with a wide range of materials, always involve a curing step, which can be chemical, photoactivated, or otherwise. Material deposition in the X, Y, and Z dimensions is controlled by actuators that precisely position the nozzle in 3D space. Printing complex geometries often requires sacrificial supports, as each new layer is built upon the previous one [44]. Most extrusion bioprinters are equipped with multiple print heads, allowing the use of various materials within a single construct. This capability enables researchers to create constructs with regional variations in biomaterials, cell types, cell densities, and signaling molecules [45]. Compared to other bioprinting techniques, extrusion bioprinters can handle higher cell densities. However, the major drawback is that the cells experience shear stress when passing through the nozzle and pressure while in the syringe before extrusion, which can reduce cell viability and function [46]. The resolution of extrusion bioprinting is determined by the nozzle size, with the minimum diameter for cell extrusion typically being 159 μm, limiting the manufacturing precision of the constructed tissue [47]. Despite its limitations in resolution and cell activity, extrusion bioprinting remains the most widely used method in tissue engineering due to its high throughput, low cost, compatibility, and versatility [48].

(2) Inkjet-based bioprinting is a non-contact printing method derived from desktop inkjet printing, where individual droplets create patterns on a substrate. During bioprinting, an energy drive deforms the printhead, causing bioink to form droplets that are sprayed onto the substrate, building the desired 3D shape by depositing multiple layers of bioink [49]. Common methods are classified into thermal and piezoelectric actuator techniques, based on how droplets are generated. In thermal technology, ink droplets are produced by heating, creating an inflated bubble that forces the ink out of a narrow nozzle onto the substrate [50]. In contrast, the piezoelectric method generates drops through transient pressure, ensuring the droplets are directional, uniform, and consistently sized [51]. However, due to the small driving force in inkjet bioprinting, high viscosity bioinks or high-density cells often clog the printhead [52]. Potential issues include nozzle clogging from high cell density, low droplet directionality, and exposure to thermal and mechanical stress during droplet formation [53].

(3) Laser-assisted bioprinting utilizes a laser as the energy source to deposit biomaterials onto a substrate. This technique typically comprises three components: a pulsed laser source, a ribbon coated with liquid biological materials, and a receiving substrate [54]. Focused laser pulses from above locally heat the bioink through the interlayer, causing a vapor bubble to rapidly expand and collapse, creating a pressure wave that propels droplets from the interlayer ribbon onto the receiving substrate [55]. During the bioprinting process, the size and frequency of droplets can be adjusted by controlling parameters, such as bioink viscosity and laser intensity. Despite its unique advantages, including high resolution, a wide variety of available bioinks, and preserved cell viability, laser-assisted bioprinting faces challenges, such as high costs, complex operation, limited 3D construction capabilities, and the need for ongoing research to improve its ability to create heterogeneous structures [56].

(4) Stereolithography bioprinting selectively solidifies a cell-laden bioink using photo-polymerization in a layer-by-layer process controlled by a moveable stage along the z-axis. A 2D pattern is projected onto the bioink reservoir, allowing the creation of complex 3D structures without a printhead moving in the x–y directions. This results in a faster bioprinting process compared to other nozzle-based bioprinters [57]. Unlike traditional extrusion printing, stereolithography does not require physical contact to manipulate individual layers. Instead, it uses controlled illumination to selectively photocrosslink liquid bioinks into solid structures, offering higher spatial resolution through precision lasers or video projectors [58]. Additionally, since no nozzle or shear force is applied to the material and cells, the printed tissue structures maintain high cellular activity [59]. However, as the cell density increases, the light transmission of bioinks decreases, making this technique unsuitable for high cell density bioinks [60].

Bioink is one of the major elements for 3D bioprinting. There are many biomaterials that can be used as bioinks. Among these biomaterials, hydrogels play the most important role in 3D bioprinting and serve as the main sources of developing bioinks for cartilage regeneration. They can be precisely modified to replicate the physicochemical properties of cartilage and provide an environment similar to that of the native ECM, which is beneficial to the proliferation and differentiation of encapsulated cells [61,62]. Currently, the hydrogel materials applied as bioinks in the field of 3D bioprinting involve natural materials, including alginate, hyaluronic acid (HA), collagen, silk fibroin (SF) [63,64,65,66], and synthetic polymers such as polyethylene glycol (PEG), gelatin methacryloyl (GelMA), polylactic acid (PLA), poly-vinyl alcohol (PVA), hyaluronic acid methacrylate (HAMA), etc. [67,68,69,70,71]. Next, we will outline the properties of the above-mentioned hydrogels as bioinks in 3D bioprinting for cartilage tissue engineering and focus on the latest research and application progress in cartilage regeneration.

### 4.1. Natural Hydrogels

The natural hydrogel is mainly based on proteins and polysaccharides of the extracellular matrix (ECM). The protein-based hydrogels include gelatin, fibrin, elastin, and silk fibroin. The polysaccharide-based hydrogels mainly include chitosan, hyaluronic acid, alginate, and chondroitin sulfate. According to the origin and relation to the recipient tissues, natural hydrogels can be divided into allogeneic, xenogeneic, and autologous hydrogel. Several hydrogels, such as hyaluronic acid, gelatine, and collagen, can simultaneously belong to all the groups above because of their sources, coming from both animal and human materials. Duo to the similar characteristics to the ECM, such as biocompatibility, biodegradability, low cytotoxicity, and low immunogenicity, natural hydrogels promote cell adhesion, growth, proliferation, and differentiation. Currently, natural hydrogels are widely used as bioinks in 3D printing for cartilage tissue engineering (Table 1).

#### 4.1.1. Alginate

Alginate is a natural polysaccharide extracted from brown algae, which is highly hydrophilic and biocompatible. It is characterized by being non-cytotoxic, cost-effective, and eliciting a low immune response [72,73]. Chemically, alginates are copolymers mainly composed of β-D-mannuronic acid (M) and L-guluronic acid (G), linked by glycosidic bonds. Usually, alginates are easy to gelatinize through crosslinking rapidly with a calcium ion solution such as calcium chloride (CaCl_2_) [74,75]. Duo to its availability and lower cost, alginates are widely applied as bioink [76]. Many studies showed that alginates have an excellent biocompatibility and non-toxicity; thus, alginates, whether used only as scaffolds or as bioprinted scaffolds with cells, can accelerate the repair of cartilage tissue [77,78,79]. Alginate itself degrades slowly and is inherently non-degradable in mammals due to the absence of the enzyme (alginase) to cleave the polymer chains.

However, alginate hydrogels are easily degraded over time *in vivo* due to the loss of calcium ions and thus are also considered to be lacking the biological stability of scaffolds. In the past decade, researchers have been exploring a variety of different methods to increase the stability and mechanical properties of alginate hydrogels to better meet the criteria for application in cartilage repair. Balakrishnan et al. demonstrated that an oxidized alginate–gelatin injectable composite hydrogel underwent self-crosslinking in the presence of borax, yielding no significant inflammatory or oxidative responses during the gelation process. This hydrogel also showed a good performance in integrating with host cartilage, facilitating encapsulated chondrocytes to exhibit enhanced glycosaminoglycan (GAG) deposition, and contributing to hyaline cartilage formation [80]. Notably, due to its promising printability and biocompatibility, there is a growing trend of mixing alginate with or chemically grafting it to other distinct materials to form alginate-based composite hydrogels that suit different biomedical application. Kundu et al. built a 3D scaffold, which was constructed using alginate hydrogel with chondrocytes and presented in the form of multi-layer deposition of polycaprolactone (PCL). The results of the *in vivo* experiment showed more COL II fiber and better cartilaginous tissue formation in the PCL/alginate/chondrocyte/TGF-β scaffold [81].

Kosik-Kozioł et al. formulated an alginate/short submicron polylactide ink, which was able to increase Young’s modulus of bioprinted scaffold and neocartilage ECM deposition [82]. Kilian et al. fabricated a 3D-printed alginate-based multifunctional bioscaffold incorporating calcium phosphate cement (CPC) and alginate-methylcellulose (algMC) in different layers to mimic the osteochondral tissue. The results showed that human chondrocytes were able to proceed chondrogenesis in both mineral-free and mineralized layers, suggesting that the layer-by-layer function in 3D bioprinting could play a pivotal role in mimicking the heterogeneous environment and structure of the osteochondral tissue [83]. In order to increase the printability of alginate materials, Olate-Moya et al. constructed a new material which is composed of photocrosslinkable alginate, gelatin, chondroitin sulfate, and graphene oxide. This material was more suitable for printing and maintained the shape of the printed scaffolds. The *in vitro* assay showed that this nanocomposite hydrogel bioink presented higher cell proliferation than pure alginate and induced chondrogenic differentiation of human adipose tissue-derived mesenchymal stem cells (hADMSCs) without the application of exogenous chondrogenic factors [84]. In addition, Schwarz et al. used an oxidized alginate–gelatin hydrogel system, consisting of alginate-di-aldehyde (ADA) and gelatin, to fabricate 3D-printed scaffolds by enzymatic and ionic crosslinking techniques. The results showed that the 3D-printed ADA-GEL hydrogels provided the suitable cultivation environment of cells for cartilage tissue engineering [85].

Yu X et al. formulated a double-layer hydrogel scaffold by using agarose as a base material, in which a sodium alginate (SA)/agarose layer was used for the repair of artificially produced subchondral bone defects, while a decellularized ECM (dECM)/agarose layer was used for the repair of articular cartilage defects. The animal experiments showed that the surface of the new cartilage tissue in the double-layer hydrogel scaffold group was closest to normal articular cartilage, with a structure similar to that of hyaline cartilage and a preliminary calcified layer. This clearly showed that in situ fabrication of an anisotropic double-layer hydrogel as a bioscaffold could promote the functional repair of articular cartilage and subchondral bone, favoring the close integration between the newly formed tissue and the adjacent host tissue [86].

Despite these advancements, there are several drawbacks associated with using alginate for cartilage tissue engineering. For example, due to the lack of adhesive ligands and the existence of negative charges, pure alginate shows minimal cell adhesion, which limits cell proliferation. Furthermore, alginate still cannot match the mechanical requirements of native cartilage tissue. To address these issues, recent studies have incorporated Arg-Gly-Asp (RGD) adhesive peptide ligands into alginate to enhance cellular adhesion and their mechanical properties [87,88]. Oxidized alginate and citric acid have also shown to be a promising strategy for enhancing cartilage regeneration, which may contribute to a versatile application in cell delivery and tissue engineering [89,90]. In addition, notable improvements in mechanical properties have been achieved by combining alginate with other biomaterials, such as gelatin or HA [91,92]. Further development of hydrogels with customized pore properties for defective cartilage is expected to meet the requirements of the ultimate clinical application.

#### 4.1.2. HA

HA is a natural glycosaminoglycan characterized by repeating disaccharide units of glucuronic acid and *N*-acetylglucosamine [93]. As one of the major components in the ECM, HA significantly enhances chondrogenesis by directly affecting the attachment, proliferation, differentiation, and migration of chondrocytes [94,95]. HA is able to bind to cell surface receptors called hyaladherins, such as CD44 (Cluster of Differentiation 44), CD168 or RHAMM (Receptor for Hyaluronan-Mediated Motility) and LYVE1 (Lymphatic-Vessel Endothelial Hyaluronan Receptor 1), which play a very important role in cell migration during the inflammatory phase of tissue repair.

In addition, aggrecan and other proteoglycans, which are the matrix components, can also act as receptors for HA and promotes chondrogenesis [96]. Due to its unique relationship with chondrocytes, HA is considered as an ideal biomaterial for cartilage repair and has been widely used in cartilage tissue engineering.

Current research efforts primarily focus on the application of HA-based injectable hydrogels and the use as 3D printing bioinks. Injectable HA hydrogels, including aldehyde HA-based, thiolated HA-based, and phenolized HA-based formulations, exhibit excellent rheology properties, being suitable for injection. With functionalization, it allows self-polymerization through free-radical polymerization and photo-crosslinking methods [97,98,99]. Wang et al. developed a double-layer scaffold based on injectable HA-SH/Col I incorporating BCP ceramic hydrogel for regenerating rabbit condylar osteochondral tissue. This HA-based hydrogel encapsulated the rabbit bone marrow MSCs (rBMSCs) and chondrocytes, promoting condylar fibrocartilage and subchondral bone formation from the upper to lower layers [100]. HA is also integrated with other biomaterials to form hybrid bioinks for 3D bioprinting. Shokri et al. reported the creation of an HA–gelatin–elastin (EGH) blend bioink, which was fabricated into a 3D-printed EGD scaffold for nasal septal cartilage regeneration. The *in vivo* experiment showed that the EGH constructs exhibited excellent interaction with the surrounding tissues, without any distinct boundaries, and promoted neocartilage formation at the defect margins [101]. Shi et al. developed a dynamic HA-based composite hydrogel with covalent crosslinked gelatin, demonstrating strong potential as an anti-oxidative bioink for articular cartilage tissue engineering. This type of composite bioink exhibited abilities to protect embedding chondrocytes from reactive oxygen species (ROS)-induced damage and effectively supported the chondrogenic differentiation of encapsulated adipose-derived mesenchymal stem cells (ADMSCs). Additionally, it enhanced the ECM production and exhibited excellent stability and compatibility during *in vivo* experiments [102]. Zhu et al. created elastin-like protein-HA (ELP-HA) hydrogels through dynamic hydrazone bonds via the reaction between hydrazine-modified ELP (ELP-HYD) and aldehyde-modified HA (HA-ALD). The hydrogels were fabricated with variable HA concentrations (1.5%, 3%, or 5%), and chondrocytes were encapsulated. The results showed that increasing the HA concentration led to a dose-dependent increase in cartilage-marker gene expression and enhanced sGAG deposition, while minimizing the undesirable fibrocartilage phenotype [103]. Recently, Lin et al. developed a visible light-activatable methacrylated gelatin (mGL) hydrogel, and further optimized mGL scaffolds by supplementing methacrylated HA (mHA). The optimized mGL/mHA at a 9:1 (%, *w*/*v*) ratio resulted in the lowest hBMSC hypertrophy and highest glycosaminoglycan (GAG) production, and the implantation of the mGL/mHA (9:1) construct improved both cartilage and subchondral bone regeneration [104]. In addition, scaffolds acting as carriers for delivering drugs or growth factors also accelerated cartilage repair. Titan et al. found that human leukocyte–platelet-rich plasma (L-PRP) or leukocyte–platelet-rich fibrin (L-PRF) delivered on an HA scaffold to a cartilage defect, using L-PRP and L-PRF in conjunction with an HA scaffold, improved biomechanical strength, enhanced cellular migration, and increased sulfated GAG (sGAG) and collagen content, compared to using an HA scaffold alone [105].

HA is emerging as a promising biomaterial applied in the cartilage tissue engineering, given its biocompatibility, biodegradation, and the favorable biomimetic environment it creates, promoting cell adhesion and proliferation for cartilage repair. Despite existing limitations in HA such as poor mechanical properties, rapid degradation, and low shape fidelity, efforts are being made to functionalize HA with specific groups and develop HA-based composite hydrogels to address these drawbacks. This ongoing effort provides novel prospects and applications in cartilage tissue engineering.

#### 4.1.3. Collagen

Collagen exists mainly in the connective tissue of mammals and is an important component of the ECM. It is widely found in the bones, tendons, muscle sheaths, and in the membranes, ligaments, cartilage, and skin of animals [106]. It accounts for 25~30% of the total protein in the animal, plays a role in supporting organs and protecting the body, and is also the most important functional protein that constitutes the intercellular substance of cells. There are many types of collagen. Generally, COL I exists in skin and bones, and Col II in cartilage. The molecular structure of collagen is characterized by a triple helix structure in the form of (glycine-X-Y)3, which makes the molecular structure very stable, and has the characteristics of low immunogenicity and good biodegradability, emerging as a promising scaffold in tissue engineering [107,108,109].

In the field of cartilage tissue engineering, COL I and COL II are the most commonly used for cartilage regeneration. Yang et al. used COL I or agarose (AG) mixed with sodium alginate (SA) to serve as bioinks and incorporated chondrocytes to construct 3D-printed cartilage tissue. The results showed that the mechanical strength was improved in SA/COL I compared to SA alone and distinctly facilitated cell adhesion, accelerated cell proliferation, and enhanced the expression of cartilage-specific genes, such as *Acan*, *Col2al*, and *Sox9*. It indicated that SA/COL I effectively suppressed the dedifferentiation of chondrocytes and preserved the phenotype [65]. In addition, collagen hydrogels encapsulated with MSCs have proven effective in supporting chondrogenesis as well, facilitating the differentiation of MSCs into chondrocytes [110,111]. Lu et al. conducted research employing both COL I and COL II hydrogels embedded with adipose tissue-derived stem cells (ASCs) for chondrogenesis. Comparative analysis revealed that COL II hydrogels significantly enhanced the chondrogenesis of ASCs by influencing cell phenotype through β1 integrin-mediated Rho A/Rock signaling, in contrast to the COL I hydrogel [112]. Another approach in the collagen–hydrogel application for cartilage regeneration is blending with other components to mimic structure and stimulate repair process. Lu et al. developed an injectable collagen-based composite hydrogel (collagen–genipin–carbon dot nanoparticles, CGN). This CGN composite hydrogel, enhanced with mechanical properties and embedded carbon dot nanoparticles, triggered the mediation of reactive oxygen species (ROS). Moreover, it played pivotal roles in promoting the chondrogenic differentiation of BMSCs and regenerating the cartilage defects *in vivo* [113]. However, directly applying collagen as a source for 3D bioink is difficult because it is hard to maintain the porous structure of printed products due to the low viscosity and weak mechanical strength of collagen hydrogel [114]. Thus, it needs to be blended with other materials or chemically modified to change its properties and make it more conducive to better printing. Shim et al. fabricated a new 3D-printed scaffold which was composed of atelocollagen and supramolecular HA. This construct, incorporated with hMSCs, showed outstanding regenerative ability for the reconstruction of an osteochondral tissue in the knee joints of rabbits [115]. Yang et al. developed a bi-phasic osteochondral tissue engineering scaffold based on a composite design which combines a closely bonded subchondral layer and a cartilage layer. The peptide/TCP/PLGA scaffolds were produced via cryogenic 3D printing to create an osteogenic platform for rBMSCs assembly and osteogenic differentiation. A thermal-responsive poly(D,L-lactic acid-*co*-trimethylene carbonate) (P(DLLA-TMC)) frame was printed on top of the subchondral layer to provide sufficient bonding strength between the cartilage layer and the subchondral layer; TGF-β1 containing Col I hydrogel was dispensed in the macropores of the shape memory P(DLLA-TMC) frame to create a local environment for rBMSCs assembly and chondrogenic differentiation. The results showed that these scaffolds had heterogeneous microstructures and gradient mechanical properties and improved the chondrogenic differentiation of rBMSCs at the cartilage layer. This study provides a facile way to produce integrated osteochondral scaffolds for concurrently directing rBMSC osteogenic/chondrogenic differentiation in different regions [116]. Yang et al. developed a collagen-based bioink integrated with the modified human-derived collagen and chitosan, which was photo-responsive and cured under UV light. Additionally, the collagen-based bioink demonstrated significant biodegradation capabilities and improved printability, allowing for the construction of well-defined 3D structures via extrusion-based printing. With its excellent biocompatibility and bioactivity, it highlights the further potential for chondrogenesis and application of articular cartilage tissue engineering [117]. Lan et al. also fabricated a novel collagen-based bioink enhanced with thiolated HA (THA) and polyethylene glycol diacrylate (PEGDA). This research demonstrated that the bioink of methacrylate-collagen (COLMA) + THA +PEGDA not only exhibited superior printability but also enhanced the cell viability and cartilaginous ECM production during *in vitro* culture. Furthermore, this bioink showed the great potential for generating autologous cartilage substitutes, offering a valuable approach to address the hyaline cartilage defects and providing a perspective for clinical therapeutic applications [118].

In cartilage tissue engineering, collagen-based cartilage scaffolds can enhance the expression of chondrogenic marker genes and promote cartilage regeneration, which effectively reduces the risk of immune rejection of the engineered cartilage. It has been demonstrated that even without using the growth factors to induce seed cell differentiation, Col II can induce the differentiation of MSCs into chondrocytes and maintain the chondrocyte phenotype [119,120,121,122]. However, the faster degradation rate and poor mechanical properties limit the application of collagen for engineered cartilage. In addition, Col II hydrogel exhibits superior chondrogenesis and cartilage repair compared to Col I hydrogel, but the newly formed tissues are often fibrocartilage rather than hyaline cartilage. These considerations should be addressed for the further applications in cartilage tissue engineering.

#### 4.1.4. SF

SF is recognized as a natural biomaterial, spun by silkworms and spiders [123]. It is composed of 43% glycine, 30% alanine, and 12% serine [124]. The SF fiber consists of bundles of interlocking nanofibrils with diameters of 30–35 nm, demonstrating a micro- and nanoscale hierarchical structure. SF is composed of a heavy chain polypeptide (∼390 kDa) and a light (L) chain polypeptide (∼26 kDa) in a 1:1 ratio and linked via a single disulfide bond [125]. SF has good biocompatibility and biodegradability. To obtain pure and soluble SF, SF needs to undergo processes such as degumming, washing, and drying [126]. Similar to the biomaterials mentioned above, SF also exhibits various characterizations, such as favorable mechanical properties, minimal immune response, desirable biodegradability, and optimized structural integration [127]. SF hydrogel is usually crosslinked by the addition of crosslinkers, such as glutaraldehyde and genipin, which might have toxic effects on cells. Therefore, some studies aim to develop a cross-linker-free silk–gelatin-based bioink formulation for cartilage regeneration [128,129,130].

Due to its exceptional biocompatibility, SF serves as an ideal 3D matrix and bioscaffold for enhancing cell adhesion, proliferation, and differentiation in tissue engineering. Moreover, owing to its derivation from natural proteins with a similarity to skin tissue, SF proves to have an outstanding capacity to interact with the extracellular matrix of chondrocytes, such as collagens and glycosaminoglycans, effectively mimicking the microenvironment conducive to chondrocyte proliferation and differentiation in bone/cartilage tissue engineering [131,132,133]. Recently, Wu et al. reported that SF could be involved in chondrocyte regulation, in combination with the growth factor (TGF-β), to facilitate rBMSCs differentiation and promote *in vivo* cartilage regeneration [134]. In addition, with good biocompatibility and biodegradation, recent studies highlighted the advantageous use of SF as an optimal supportive material, combined with other biomaterials to create composite scaffolds that facilitate cartilage regeneration. Zhang et al. used a silicate nanoclay in a novel enzymatically crosslinking method to produce an SF-Laponite (LAP) composite hydrogel, exhibited enhanced biocompatibility, biodegradation, and mechanical properties. This composite hydrogel was found to promote hyaline cartilage formation and enrich ECM synthesis in subchondral defect areas [135]. Shi et al. utilized 3D bioprinting technology to create a silk fibroin–gelatin dual-bioscaffold for cartilage regeneration, demonstrating a promising balance between biodegradation and mechanical properties. These 3D-printed constructs also provided a suitable bioenvironment for BMSCs proliferation and mechanical protection before the formation of neocartilage [136]. Pan et al. developed an antioxidant scaffold by incorporating SF and kartogenin (KGN)-loaded liposomes (SF-Lipo@KGN). *In vitro* experiments revealed that the SF-Lipo@KGN scaffolds exhibited excellent biocompatibility, as evidenced by the enhanced cell adhesion, migration, and proliferation of chondrocytes. *In vivo* experiments demonstrated the effective promotion of articular cartilage regeneration by the SF-Lipo@KGN scaffolds, which enhanced the extracellular matrix anabolism and restored the intrinsic redox homeostasis. This study showed that the biomimetic KGN-loaded scaffolds can restore cartilage redox homeostasis, indicating promising prospects for cartilage tissue engineering [137]. Chakraborty et al. fabricated an arch-like construct using two types of bioinks, gelatin methacryloyl (GelMa) and silk fibroin–gelatin (SF-G). The data showed that the bioprinted SF-G constructs displayed increased the proliferation of the encapsulated human MSCs and better formed the fibrous collagen network and chondrogenesis compared to the GelMA constructs due to the role of SF-G in regulating the Wnt/β-catenin and TGF-β signaling pathways [138]. Wu et al. developed a series of injectable hydrogel composite scaffolds (SF-GMA/LKP) by combining Ac-LIANAKGFEFEFKFK-NH2 (LKP, a self-assembled peptide nanofiber hydrogel that can mimic the function of TGF-β1) with glycidyl methacrylate (GMA)-modified silk fibroin (SF). The results demonstrated that the SF-GMA/LKP10 and SF-GMA/LKP20 composite scaffolds had the best effect on neocartilage and subchondral bone reconstruction due to the interaction between LKP and SF-GMA, which prolongs the duration of action of LKP. Hence, this composite hydrogel scaffold can be used for high-quality cartilage repair [139]. Yan et al. established and evaluated a SF-based bioink composed of hydroxypropyl cellulose methacrylate (HPCMA) to form a composite bioink, SF-HPCMA, showing excellent biocompatibility and rheological properties, suitable for 3D bioprinting applications. The printed SF-HPCMA bioscaffolds exhibited a compression modulus comparable to that of normal articular cartilage, indicating robust mechanical strength. Additionally, during *in vitro* culture, rBMSCs encapsulated in the SF-HPCMA presented great biocompatibility and chondrogenic differentiation. Furthermore, *in vivo* experiments demonstrated the high efficacy of SF-HPCMA in repairing rabbit articular cartilage defects, indicating its therapeutic potential for clinical applications [140].

In addition, a decellularized extracellular matrix (dECM) was applied as a natural biomaterial similar to SF for tissue regeneration. Zhou et al. constructed dECM-SF hydrogel by a second crosslinking method of γ irradiation and ethanol induction. This hydrogel showed an excellent porous structure and possessed swelling capacity, surface wettability and no cytotoxicity. The results demonstrated that dECM-SF could promote the chondrogenic differentiation of adipose-derived stromal cells (ADSC) and engineered cartilage formation [141].

Despite the fact that SF possess great biocompatibility, low immunogenicity, and slow degradation, the SF materials and scaffolds formed from SF for tissue engineering sometimes show biological and mechanical characteristics that do not match well with those of the surrounding body environment. In addition, in order to obtain the best results of cartilage repair, it is also important to determine the rate of degradation of the material and the degradation pathway, especially for long-term clinical applications. The resolution of these issues will help to further expand the application of the SF material in cartilage tissue repair and regeneration.

### 4.2. Synthetic Hydrogels

Although natural hydrogels possess many advantages, such as low immunogenicity, great biocompatibility, a favorable biomimetic environment and cell growth, they also have drawbacks, including low mechanical properties, rapid degradation, difficulty in shape maintenance, and the need to modify the chemical properties. Therefore, in order to overcome these shortcomings and disadvantages, biopolymers are used in biofabrication, and synthetic hydrogels will offer numerous advantages, including the capacity for photopolymerization, customizable mechanical properties, tunable degradation rate, and precise control over batch–batch variation [142]. Synthetic hydrogels can be modified for specific applications by incorporating biomolecules or diverse functional groups. Compared with natural hydrogel, synthetic hydrogels usually own lower cell adhesion abilities due to the lack of adhesion sites. However, this disadvantage can be compensated by chemical or bioactive modifications. Moreover, synthetic hydrogels can mimic and display the physical attributes of biological tissues such as joints, contributing to better utilization in osteochondral repair by adjusting the polymer chain lengths, molecular structure, and molecular weight [143]. The following discussions will be focused on the various synthetic hydrogels and their applications in cartilage repair (Table 2).

#### 4.2.1. PEG-Based Hydrogel

As an FDA-approved synthetic polymer, polyethylene glycol (PEG) assumes a vital role in the field of biomedical applications such as tissue engineering and drug delivery, particularly in cartilage regeneration, owing to its excellent biocompatibility, minimal immunogenicity, and resistance to protein adsorption [144]. During the biomedical applications of PEG, the fabrication approach for PEG materials significantly influences the physicochemical and biological properties of hydrogels. In fact, the production of PEG hydrogels primarily involves three cross-linking methods: (1) the radiation of linear or branched PEG polymers; (2) the free radical polymerization (FRP) of PEG acrylates, (3) specific chemical reactions, such as click chemistry and enzymatic reactions [145].

In recent years, photopolymerization, utilizing light to convert liquid PEG macromer solutions into solid hydrogels under physiological conditions, has been developed and plays a unique and primary role in forming PEG hydrogels for cartilage repair. This technique provides advantages in creating hydrogel scaffolds in situ with precise spatial and temporal control, enabling the development of diverse 3D structures encapsulating cells and biological agents [146]. Ravi et al. demonstrated the creation of a highly tailored hydrogel scaffold through 3D printing, utilizing the photopolymerization capability of PEG diacrylate (PEGDA), a modified form of PEG macromers. This PEGDA hydrogel, with optimal mechanical properties and a customizable structure, was adept at consistently encapsulating umbilical cord-derived mesenchymal stem cells (UMSCs) and cobalt nanowires. The cobalt nanowires exhibited the potential to induce a hypoxia-inducible factor-1α (HIF-1α)-associated chondrogenic effect by inhibiting HIF-1α degradation. This system not only provided a conducive microenvironment for UMSCs but also effectively mimicked hypoxic conditions and facilitated UMSCs differentiation into the chondrocyte lineage without the use of growth factors [147]. Similarly, Bandyopadhyay et al. also developed a composite hydrogel using PEGDA and silk methacrylate, capable of recreating the biological niche of chondrocytes. With appropriate mechanical properties and degradation rate, the composite hydrogel demonstrated an ability to enhance Col II and GAG deposition, suggesting its potential to promote cartilage regeneration [148].

Although photopolymerization stands out as a fast and convenient method for creating PEG hydrogels, significant apprehensions emerge concerning cell exposure to light, particularly in the ultraviolet (UV) range. This exposure has the potential to induce mutagenic and cytotoxic effects on cells [149]. Fortunately, PEG exhibits both linear and branched (multi-arm or star) structures. The foundational PEG structure with hydroxyl end groups can undergo diverse conversion into various functional groups, including methyloxyl, carboxyl, amine, thiol, azide, vinyl sulfone, and acetylene, either symmetrically or asymmetrically, offering versatility for hydrogel formation without UV [150]. Wang et al. discovered and found that, at overlap concentration, 4-arm PEG functionalized with vinyl sulfone group reacted with a short dithiol crosslinker, resulting in the formation of a PEG hydrogel characterized by remarkable compressive strength and a Young’s modulus resembling that of cartilage. This distinctive mechanical property enabled the hydrogel to enhance cartilage repair by closely mimicking the chondrocyte microenvironment in nature, which is essential for facilitating the encapsulated cells in promoting the secretion of the ECM and maintaining their typical phenotypes [67,151].

In addition, PEG hydrogels have been extensively utilized as matrices to control drug delivery and as vehicles for cell delivery for tissue regeneration because of their optimal biodegradability, hydrophilia, body-tissue compatibility, and the ability to mimic the mechanical structure of cartilage. The adaptability of PEG macromer chemistry, coupled with its outstanding biocompatibility, has catalyzed the creation of numerous strategically designed hydrogel systems for applications in regenerative medicine. However, PEG hydrogels are relatively biologically inert. Thus, bioactive modifications such as the incorporation of RGD peptides are usually used as complementary associates to fabricate engineered PEG hydrogel in order to have better therapeutic effect and overcome bio-inertness [152]. For instance, Yang et al. designed a modified PEG hydrogel crosslinking with cysteine–arginine–glycine–aspartic acid (CRGA, a cell adhesion peptide) via the Michael Addition Reaction. Due to the improved cell adhesion capability and bioactivity, CRGA-conjugated PEG facilitated the chondrogenic differentiation of peripheral blood mesenchymal stem cells (PBMSCs) and promoted M2 macrophage polarization, which is crucial for the wound healing process [153]. In short, the composite hydrogels have sufficient mechanical properties required for articular cartilage repair and provide a positive chondrogenesis environment.

#### 4.2.2. GelMA-Based Hydrogel

Gelatin, a protein found in the ECM and derived from denatured collagen, is a natural material with biocompatible and biodegradable properties. Although gelatin itself forms a gelatinous structure, its relatively low melting temperature (31.7–34.2 °C) limits its direct application as a cell carrier [154]. While pure gelatin can be solidified using glutaraldehyde, this method is cytotoxic, time-consuming, and difficult to precisely control. To address this, the addition of stabilizing components is necessary for *in vivo* applications. In 2000, Van Den Bulcke et al. discovered that gelatin methacryloyl (GelMA), a modified gelatin, could be crosslinked via photopolymerization in the presence of methacrylate, resulting in a hydrogel with improved thermostability and sustained biocompatibility [155].

GelMA is produced by reacting gelatin with methacrylic anhydride (MA). Briefly, this reaction replaces numerous amino groups on gelatin’s side chains with methacryloyl groups and forms a synthetic material. GelMA’s photocrosslinking ability, which is facilitated by the methacryloyl groups, allows for immediate crosslinking under UV light and produces hydrogels with excellent thermostability. GelMA hydrogels maintain the biocompatibility and degradation properties of gelatin and make them more suitable for biological applications [156]. Tissue engineering aims to establish an optimal microenvironment for cell growth, and GelMA hydrogels can be tailored to meet various requirements by adjusting the hydrogel concentration, functionalization degree, UV intensity, and additives [157]. Miri et al. found that the permeability of GelMA varied widely through adjusting the hydrogel concentration and crosslinking density. Therefore, GelMA is an appropriate vehicle for a variety of drugs and molecules because of its tunable and elastic permeability range [158]. Although GelMA hydrogels are widely used in 3D cell culture, their practical biomedical applications, especially for load-bearing tissue repair, are hindered by low mechanical strength, rapid degradation, and an inability to accurately mimic native tissues. To overcome these limitations, one strategy involves integrating GelMA with other materials, such as natural substances, synthetic polymers, and nanoparticles, to form hybrid or composite hydrogels, leveraging the advantages of multiple components. Paul et al. incorporated glycol chitosan (GC), a water-soluble material derived from chitosan, with GelMA to fabricate a composite material which can be crosslinked by tris (2,2′-bipyridyl) dichlororuthenium (II) hexahydrate ([RuII(bpy)3]2+) and sodium persulfate (Ru/SPS). With the addition of GC, this composite hydrogel showed significantly improved adhesive capability and enhanced compressive modulus, which were more comparable to natural cartilage. Furthermore, the synergic effect of the modified crosslinking method and composite material allowed the hydrogel to exhibit increased sustainability and a suitable degradation period [159]. Liu et al. developed a composite hydrogel CM-KGN@GelMA, which contained Cytomodulin-10 (CM-10, a TGF-β1 short peptide) and Kartogenin (KGN, a small molecule). The results showed that the hydrogel CM-KGN@GelMA had a synergistic effect on promoting the chondrogenesis of BMSCs by upregulating the expression of mRNA and protein levels of RUNX1 and SOX9 *in vitro*, and it promoted osteochondral defect repair *in vivo*. This suggests that the modified hydrogel CM-KGN@GelMA acts as a potential scaffold for osteochondral defect regeneration [160]. In addition, Sun et al. showed that GelMA could be mixed with different components to print a bilayer porous hydrogel scaffold with different modulus and composition in the upper and lower layers through 3D printing technology. The upper scaffold added black phosphorus (BP) and human umbilical cord MSCs (hUMSCs) exosomes (exos) in GelMA, which had a relatively lower elastic modulus and was conducive to the differentiation of BMSCs into chondrocytes. In the lower scaffold, in addition to BP and hUMSCs exos, β-tricalcium phosphate (β-TCP), which had osteoconductive and osteoinductive effects, was added to GelMA. The *in vitro* data showed that the bilayer scaffolds promoted osteogenesis and chondrogenic differentiation, respectively. The *in vivo* data also indicated that the 3D-printed bilayer GelMA composite scaffold has a repair effect close to normal tissue [161]. In addition, improving the performance of GelMA-based hydrogels involves not only chemical composition engineering through the introduction of new components but also the control and design of physical microstructures to closely emulate the physical cues of the native tissue microenvironment. Yin et al. created a 3D printable gel-in-gel hydrogel that combines GelMA and alginate hydrogel, integrating alginate/GelMA microspheres composed of chondrocytes and TGF-β1. Leveraging the benefits of GelMA composite microspheres, which include exceptional encapsulation capability, micron-scale size, and customizable structural options, this hydrogel, embedded with microspheres, can be printed and transplanted into the subcutaneous tissue of rats. The transplantation results demonstrated an improved outcome in cartilage repair, presenting an alternative approach for future research on the treatment of osteoarthritis (OA) [162].

**Figure 4:**
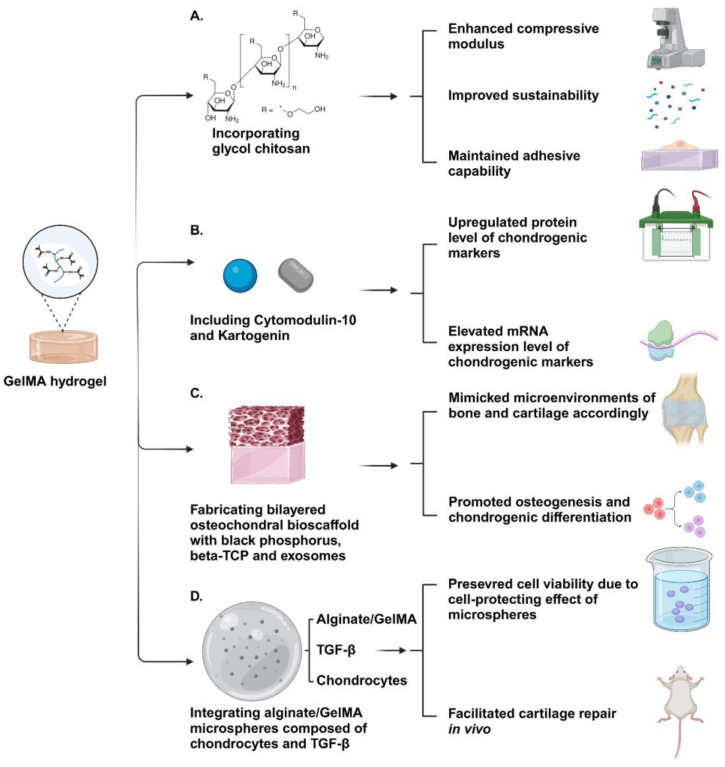
Schematic illustration of GelMA-based hydrogels. (**A**) The GelMA–glycol chitosan hydrogel exhibited excellent mechanical properties and an appropriate degradation period while maintaining high adhesive capability. (**B**) The increased expression of chondrogenic markers in encapsulated BMSCs suggested that Cytomodulin-10-modified GelMA with Kartogenin holds significant potential for enhancing cartilage regeneration. (**C**) The bi-layered GelMA-based hydrogel containing black phosphorus, Beta-TCP, and exosomes effectively recapitulated the microenvironments of bone and cartilage, facilitating the osteogenesis and chondrogenic differentiation of encapsulated MSCs, respectively. (**D**) The bioink containing microsphere-embedded chondrocytes demonstrated enhanced cartilage regeneration outcomes through transplantation in animal models, benefiting from its biomimetic encapsulation environment and multiscale structure. Created with BioRender.com.

In conclusion, GelMA plays a unique and irreplaceable role in biomedical applications because of its advantageous biocompatibility, bioactivity, and permeability (Figure 4). Although its mechanical properties are not completely analogous to those of natural cartilage, new developments on exploring the methods of modification on GelMA, including the incorporation of new components and adjustments to the microstructure of GelMA, are promising in circumventing its practical limitations for cartilage regeneration applications.

#### 4.2.3. PLA- and PLA Copolymer-Based Hydrogel

Polylactic acid (PLA), a semi-crystalline polymer with a gradual crystallization process, distinguishes itself in the field of biomedical applications owing to its compatibility with host tissues, hydrophobic properties, ease of processing, and ability to biodegrade [163]. Primarily derived from lactic acid (LA) through natural resource fermentation or diverse polymerization methods, PLA requires no secondary intervention. The resulting degradation products, namely water and CO_2_, pose no harm to the human body [164]. These exceptional characteristics position PLA as an outstanding biomaterial for pharmaceutical and biomedical applications, including the development of drug delivery systems [165].

The intermolecular forces governing PLA chains involve hydrogen bonding, hydrophobic, and ionic interactions. While the inherent hydrophobicity is advantageous for trapping drug-loaded nanoparticles, it poses a challenge in biomedical applications utilizing hydrogel networks. Although it aids in the capture of nanoparticles through the mononuclear phagocyte system, the hydrophobic nature impedes drug efficiency *in vivo* [166]. To overcome the limitations of hydrogel formation and drug delivery, modifications to PLA attributes can be achieved through copolymerization or crosslinking with various hydrophilic polymers. The copolymerization adjusts the amphiphilic behavior of PLA, as well as its physical and mechanical properties, enabling the development of PLA-based hydrogels. Furthermore, the inclusion of hydrophilic polymers in copolymerization enhances PLA degradation [167]. Tamai et al. fabricated biodegradable PLA-PEG hydrogel incorporating interconnected porous hydroxyapatite (IP-CHA) and recombinant human bone morphogenetic protein-2 (rhBMP-2). According to the result of subchondral implantation in rabbits, the repair progress of cartilage defects with PLA-PEG hydrogel carrying rhBMP-2 and IP-CHA was significantly promoted within 3 weeks. Moreover, the trauma was fully recovered within 6 weeks, showing that the combination of PLA-PEG hydrogel, rhBMP-2, and IP-CHA significantly enhances the healing process of articular cartilage [168]. Copolymerization also occurs between PLA and PGA via random ring-opening polymerization (ROP) to formulate PLGA, which is favorable for cartilage tissue engineering due to enhanced mechanical properties and excellent cell proliferation capability [169]. By taking advantage of Schiff base linkage, a category of reactions in click chemistry, Li et al. conjugated PLGA with PEG covalently and manufactured a PLGA/PEG composite hydrogel. Owing to its exceptional biocompatibility and mechanical properties, the proliferation of encapsulated BMSCs was promoted, accompanied by upregulated chondrogenic genes of cells and enhanced GAG secretion [170]. Not only its outstanding biocompatibility, but also its great permeability makes PLGA-based hydrogel attract researchers’ attention. Xu et al. utilized leached PLGA hydrogel with high porosity as an optimal vehicle to load xanthohumol, which is a plant-extracted anti-inflammatory molecule, to fabricate composite hydrogel with a minimal immunogenic effect. The subcutaneous transplantation result demonstrated that the pro-inflammatory factors of BMSCs cultured in the hydrogel, such as interleukin 1β (IL-1β) and tumor necrosis factor α (TNF-α), were downregulated, while Col II deposition was enhanced, indicating that the designed material can induce an anti-inflammatory effect and support cartilage repair [171].

In clinical applications, PLA stands out as one of the most prevalent biodegradable polymers. Despite the hydrophobic nature of these polymers and their lack of functional side groups for chemical crosslinking, their optimal biocompatibility, particularly in the form of PLA-based hydrogels, proves invaluable. Through strategic integration with other polymers, PLA-based hydrogels continue to play essential roles in the field of cartilage tissue engineering.

#### 4.2.4. PVA-Based Hydrogel

Poly-vinyl alcohol (PVA) is created through the alkaline hydrolysis of poly-vinyl acetate, demonstrating notable properties, such as exceptional biocompatibility, suitable biodegradation rate, and non-toxicity [172]. Its outstanding hydrophilicity and chemical stability enable it to endure extreme pH and temperature conditions, while its semicrystalline structure facilitates the efficient passage of oxygen and nutrients to cells [173]. Given its tensile strength, which is relatively comparable to that of human articular cartilage, considerable attention has been directed towards PVA and its applications in biomedicine, particularly in the development of scaffolds for drug delivery and cartilage repair [174].

PVA-based hydrogels, with biomimetic mechanical properties resembling natural cartilage, form colloidal dispersions with 3D network structures through either physical or chemical crosslinking. These hydrogels are capable of providing an appropriate biological niche for embedded chondrocytes or stem cells [175]. Zhu et al. designed a PVA hydrogel incorporated with phosphate glass fiber (PGF) to reinforce the mechanical properties of the material. Multiple tests of mechanical performance indicated that the tensile and compressive strengths of the PGF-PVA composite hydrogel are comparable to those of natural cartilage tissue. Additionally, the hydrogel network of the PGF-PVA composite material promotes ion exchange efficiency through ion flow intervention, leading to enhanced chondrocyte recruitment and proliferation [176]. As a cartilage bionic material, PVA can also be combined with other components, forming bilayer or biphasic hydrogel, to resemble osteochondral tissue with complex structure and composition. Yao et al. used a self-made mold to control the distribution of pore-forming reagents on the material and successfully developed β-TCP/PVA dual-layer hydrogel in which only half of the gel was highly porous, mimicking the distinct microenvironments of the subchondral bone and cartilage, respectively. According to the outcome of the co-culture with chondrocytes and synovium MSCs (SMSCs), the subchondral layer with high porosity promoted cells invasion and reinforced the differentiation of SMSCs into specific lineage, while the upper dense cartilage layer of the hydrogel supported chondrocyte attachment, showing that the biomimetic double layer β-TCP/PVA provided considerable biological functionality [177]. In addition, its suitable biodegradation ability and excellent biocompatibility position PVA as a suitable candidate polymer for loading drugs or biomolecules with sustained release times and localized release areas. Bolandi et al. used an alginate/PVA composite hydrogel as a vehicle to incorporate alginate sulfate microbeads carrying platelet-rich plasma (PRP), which contains various chondrogenic growth factors. With its constant localized release capacity, the drug loading system effectively promoted the proliferation of embedded adipose-derived MSCs (AMSC) and enhanced the deposition of GAGs. Furthermore, the results demonstrated that the expression of chondrogenic marker genes, including SOX9, Aggrecan, and collagen type II, were significantly upregulated, indicating that the releasing system had great potential for regeneration of cartilage tissue [178].

Looking ahead, the evolution of PVA hydrogels may progress towards a smart and patient-specific direction. Innovative preparation methods, such as 3D printing and in situ gelation have the potential to offer personalized solutions for osteochondral defects by delicately reconstructing the cartilage surface [179]. However, similar to other synthetic materials, PVA faces a common limitation: its limited cell adhesive capability and insufficient cell ingrowth [180]. To enhance its biological activity and physicochemical properties, PVA is often combined with natural and synthetic polymers to form composite materials. Various techniques of bioactive modifications on PVA, including the binding of bioactivity-associated peptides, polysaccharides, or growth factors, are also commonly employed to facilitate practical utilizations of PVA.

#### 4.2.5. HAMA-Based Hydrogel

HA is a type of GAG widely present in various tissues, especially in cartilage [181,182]. Although pure HA plays a crucial role in the cartilage matrix by inhibiting close mesenchymal cell–cell interactions, facilitating cellular migration, and regulating chondrogenic genes and growth factor signaling pathways, such as TGF-β and BMPs, its application in bioprinting for cartilage repair is restricted due to its unfavorable degradation rates and mechanical properties [183,184,185]. To overcome these limitations, HA methacrylate (HAMA) was developed by reacting HA with excess methacrylic anhydride. With its methacrylate feature [186], HAMA can crosslink with polyamino acids through photopolymerization, resulting in a hydrogel with highly adjustable mechanical properties and enhanced printability. This modification retains the essential attributes of natural HA, such as biocompatibility, lubrication, and viscoelasticity, while improving its printability and effectiveness for cartilage repair.

The creation of a hydrogel from HAMA involves the covalent crosslinking of the methacrylate polymers. Radical polymerization of the polymer-bound (meth) acrylate groups can be initiated either thermally or by exposure to light [187]. In the latter case, a small amount of initiator is introduced to the polymer solution to generate sufficient radicals, allowing crosslinking to occur upon UV irradiation. Generally, the preferred method is the light-triggered initiation of crosslinking, and various photo initiators have been developed to release radicals when exposed to different light wavelengths. The efficiency of these light-initiated reactions is influenced by factors such as the light wavelengths and intensity used, the type and concentration of the photo initiator, and the degree of methacrylation on HA [188]. By fine-tuning these parameters, the mechanical characteristics of HAMA can be precisely adjusted to replicate the biological environment of cartilage tissue, making it suitable for bioprinting in cartilage regeneration applications. Lam et al. conducted tests on the printability of HAMA bioink and GelMA bioink, revealing that both materials could be patterned to form cartilage-like models using stereolithographic printing methods. The results showed that chondrocytes encapsulated in the printed HAMA models retained their phenotype and secreted cartilage-specific ECM after *in vitro* incubation. This underscores the successful mimicry of the native ECM function by HAMA bioink and provides a suitable biological niche for chondrocytes. Furthermore, the HAMA hydrogel model also presents significant potential for constructing highly adaptable cartilage scaffolds for clinical applications [189]. In addition, HAMA has the ability to enhance chondrogenesis by supplying cells with biochemical signals to regulate their morphology and proliferation. Martyniak et al. introduced a high-throughput and noninvasive method called the human chondrocyte Col2a1 Gaussian luciferase reporter system (HuCol2gLuc) to assess Col II production. Through this approach, they determined that the inclusion of HAMA in the GelMA composite bioink significantly elevated the expression of Col II in the incorporated chondrocytes when compared to a bioprinted construct using pure GelMA. The results demonstrated that the incorporation of HAMA, which contained integral GAGs from the cartilage ECM, enhanced the formation of cartilage [190]. This indicates that HAMA hydrogel holds promise as an appealing candidate for cartilage regeneration. Kesti et al. developed a novel material to be used as bioink by blending the temperature-sensitive polymer poly (N-isopropylacrylamide)-grafted hyaluronan (HA-pNIPAAM) with HAMA. Taking advantage of the natural properties of the original materials, the new material is characterized by rapid cross-linking, print-molding, and long-term mechanical stability. Therefore, it is more suitable for bioprinting [191]. Poldervaart et al. modified HA with methacrylate groups, followed by the addition of a photo initiator, which leads to polymerization upon UV-exposure, resulting in network formation. The results of the physicochemical property analysis indicated that it increased stiffness and was resistant to degradation, while maintaining good biocompatibility [192].

Boasting a swift photosensitive response, rapid gelation speed, and consistent hydrogel performance, the HAMA hydrogel emerges as a viable 3D printable biomaterial suitable for crafting customized scaffolds, tailored to address cartilage defects. Simultaneously, the presence of HA in HAMA bestows it with significant potential to foster chondrogenesis, ultimately contributing to enhanced repair of cartilage by orchestrating the regulation of chondrogenic genes and facilitating physical cell condensation. These attributes have drawn increasing attention, positioning HAMA in a distinctive role within the realm of cartilage regeneration. Nonetheless, similar to many synthetic materials, the therapeutic impact of HAMA hydrogel for cartilage repair is hampered by the absence of cell adhesion sites. In most instances, a synergistic combination with other materials possessing exceptional cell adhesion capacity such as GelMA, or through conjugation with peptides, becomes imperative to engraft the HAMA hydrogel with enhanced capabilities for cell adhesion, proliferation and cartilage ECM deposition.

#### 4.2.6. Methylcellulose-Based Hydrogel

In recent years, cellulose (a natural polysaccharide) has garnered significant attention due to its notable properties, including excellent gel formation, non-toxicity, high biocompatibility, biodegradability, and post-printing stability [193]. Cellulose is easily transformable into various derivatives, and each owns distinct characteristics and functionalities, suitable for diverse applications, such as cartilage regeneration. Among these derivatives, methylcellulose (MC) stands out as one of the simplest, being achieved by partially substituting the hydroxyl groups of cellulose with methoxy groups. With a suitable degree of substitution, MC gains water solubility and exhibits a tunable lower critical solution temperature (LCST) behavior. This sets it apart from native cellulose, as it undergoes a reversible sol–gel transition with an increase in temperature [194]. The distinctive LCST behavior enables aqueous MC solutions to transform into turbid gels when heated beyond a certain temperature and regains transparency and flow properties upon cooling [195]. The suggested mechanism behind the gelation induced by heating comprises a two-step process. Initially, at lower temperature, MC chains create “bundles”, owing to unsubstituted cellulose regions. As the temperature increases, some of these bundles undergo hydration, revealing methylated repeat units to water. With a further rise in temperature, the hydrophobic strands connected to different bundles facilitate the development of crosslinks, resulting in mechanical stiffening and the formation of the gel [196].

MC exhibits exceptional thermosensitive gelation characteristics, rheological attributes, non-toxicity, biocompatibility, and a structurally uncomplicated nature. It functions as an inherently functional and physically crosslinked water-based matrix or scaffold for applications in cartilage engineering. The stiffness of this material can be effortlessly tuned from a viscous fluid to sturdy, self-supporting gels by adjusting the concentration of MC or the ambient temperature. In addition, MC-based hydrogels have also been utilized as injectable carriers for delivering chondrogenic cells to damaged cartilage [197,198,199]. In comparison to the nonresponsive hydrogels being studied for cartilage regeneration, MC-based hydrogels enable easy and uniform cell loading in their sol state (T < 37 °C), facilitating delivery to the host tissue. The sol–gel transition of MC-based hydrogels upon heating (T ∼ 37 °C) enhances in situ retention, setting them apart in the realm of cartilage repair. Leveraging a dispersion approach, Cochis et al. achieved the successful fabrication of an MC-based hydrogel exhibiting solution-to-gel transition around 37 °C and minimal bulk degradation. At room temperature, MSCs were homogeneously dispersed and encapsulated within the MC solution. Subsequently, the blend of MSCs and MC was introduced into a porous polyurethane scaffold and subjected to cultivation in a chondrogenic conditioning bioreactor, providing the cells with suitable mechanical stimuli. Upon elevating the incubation temperature, the MC hydrogel underwent gelation, forming a polyurethane-MC composite with favorable cell retention. After 21 days of *in vitro* incubation, a positive chondrogenic effect was evidenced by elevated expressions of chondrogenic genes and the detection of GAG and Col II production through histological analysis. The expression ratio between COL II and COL X was notably high, indicating a delayed hypertrophy of differentiated chondrocytes. These findings collectively confirmed that, under appropriate mechanical stimulation, the MC-based hydrogel serves as a suitable vehicle for MSC retention, fostering load-induced chondrogenesis while mitigating the hypertrophic effects of chondrocytes [200]. Furthermore, owing to its distinctive capability for thermal crosslinking, MC hydrogels, which can undergo gelation without the involvement of chemicals or potentially detrimental UV exposure, are harnessed in bioprinting applications for cartilage engineering. Hodder et al. detailed the utilization of a sterilized blend of alginate and methylcellulose through autoclaving or UV-irradiation, achieving precise strand deposition and yielding stable scaffolds, with the desired 3D geometry, for cartilage tissue regeneration. Bovine primary chondrocytes were incorporated into the alginate–methylcellulose blend, and the resulting bioink was employed for bioprinting. The observed high number of viable cells embedded and the enhanced production of the proteoglycan matrix following culture demonstrated the promising potential of the alginate–methylcellulose bioink in the field of cartilage tissue engineering [201].

In summary, it has been demonstrated that synthesizing MC-based hydrogels is relatively straightforward, and their mechano-rheological behavior is highly adjustable by manipulating the concentration of materials and ambient temperature. The unique thermal responsiveness of MC-based hydrogels has led to their widespread use as in situ gelling systems, 3D bioinks, and intelligent culture surfaces. Despite promising results observed in both *in vitro* and *in vivo* studies, the primary role of the plant-derived MC seems to be more centered on providing structural support, given its relatively limited functional or biological advantages in the resulting hydrogel matrix. Therefore, for the clinical application of MC-based hydrogels in cartilage repair therapies, further research and advancements in material development are still necessary.

#### 4.2.7. Polyurethane-Based Hydrogel

Enhancing the field of regenerative medicine for cartilage repair relies on the utilization of qualified biomaterials capable of restoring or repairing damaged bodily tissue while withstanding significant joint loads. Therefore, various factors, including a material’s physical attributes, structure, chemistry, degradation, and erosion mechanism, are crucial considerations when selecting biomaterials for cartilage-related applications [202]. Polyurethane (PU), an organic polymer composed of numerous organic molecules linked via urethane linkages, is highly regarded for cartilage repair due to its outstanding biocompatibility and mechanical properties, including high compliance, fatigue resistance, elasticity, and other structural attributes [203].

Bayer and colleagues first synthesized PU in the 1930s [204]. Typically, PU is synthesized through a polyaddition reaction involving diisocyanates and diols. Under catalyst-free conditions, the alcohol’s nucleophilic center adds to the isocyanate group’s electrophilic carbon, forming the urethane bond through hydrogen transfer to the nitrogen [205,206]. PU consists of alternating soft and hard segments, with the former providing elasticity and low-temperature resistance and the latter contributing strength through hydrogen bonding, involving urethane links [207,208]. The composition, ratio of hard-to-soft segments, or molecular mass variations lead to different polymer properties and hardness, allowing PU to achieve stiffness and physical properties comparable to natural tissues by adjusting the synthesis process. Similarly, PU is frequently employed as a bioink due to its favorable mechanical properties and flexibility. Hung et al. introduced a direct ink writing approach to create a PU and HA composite bioink for cartilage engineering. The fabricated scaffolds, generated without the use of heat, organic solvents, or additional crosslinkers that could potentially affect the scaffold’s biological functionality, exhibited a promising compression modulus and recovery under significant strain. From a biological perspective, the bioink demonstrated effective drug delivery by consistently loading bioactive ingredients such as growth factors (TGFβ3) and the small molecule drug Y27632, which is an inhibitor for Rho-associated coiled-coil containing protein kinase (ROCK). By releasing these molecules in a controlled manner, the bioink facilitated the self-aggregation and chondrogenic differentiation of encapsulated MSCs. The results from rabbit knee transplantation showed that the cells seeded in the scaffolds successfully produced a cartilage-specific ECM, indicating the material’s significant potential for cartilage regeneration [209]. Shie MY et al. developed water-based 3D printing using photosensitive materials for customized cartilage tissue engineering with digital light processing technology. This manufactured scaffold demonstrated better biocompatibility and mechanical properties similar to those of articular cartilage. In addition, it can be further adjusted by 3D reconstruction and morphologically fit the shape of the cartilage defect at the injured site, providing a more efficient method and also facilitating the long-term growth of engineered cartilage tissue [210].

Further, Chen YW et al. demonstrated through experimental design that they explored the optimal parameters of a suitable biological scaffold modified with light-curing materials, which could facilitate the adhesion and growth of stem cells. The results showed that the optimized cartilage scaffold exhibited good cell adhesion and maintained cell viability, and thus it could be used as a favorable material for the precise procedures of cartilage tissue engineering [211].

Beyond 3D bioprinting applications, the direct biological interactions between chondrocytes and PU hydrogel have been already explored. Grad et al. seeded calve-derived chondrocytes in the PU scaffold and observed that over a span of 42 days, representing a relatively prolonged period of *in vitro* incubation, the PU scaffold provided proper mechanical loading and stimuli, leading to a progressive increase in GAG and Col II deposition. However, transcriptional analysis revealed a decrease in aggrecan (ACAN) and procollagen II expression, along with an increased level of procollagen I. This suggested that over time, chondrocytes in the PU scaffold still underwent dedifferentiation. Thus, despite PU’s promising mechanical properties, it is not a universal solution for cartilage applications, and further comprehensive studies are needed to modify and enhance the functionality of PU-based hydrogels [212].

As stated above, PU stands out among synthetic and natural biodegradable polymers for its exceptional mechanical and physical properties, which align with human tissues. However, the consideration of PU biodegradation, similar to that of other biomaterials, is essential. The adjustment of properties can be achieved by varying the chemical composition, ratio of hard-to-soft segments, and molecular mass. To enhance PU biodegradation, combining these polymers with other biomaterials, particularly polyols such as PEG and PCL, can yield non-toxic degradation products. In addition, addressing challenges related to cell survival on scaffolds is also crucial due to low vascularization, limiting the therapeutic effects of PU-based hydrogels. Further research and development should focus on overcoming cell survival issues to design PU-based hydrogels that provide chondrogenic cells with a conducive environment for proliferation and differentiation.

## 5. Conclusions, Challenges and Prospects

Three-dimensional bioprinting technology has been widely used in the study of articular cartilage repair or regeneration. Hydrogels, both naturally derived and chemically synthesized, can be optimized to mimic the properties of articular cartilage based on their biochemical and biomechanical properties, making them important resources for 3D printing bioinks. In order to further improve the repair and regeneration capacity of articular cartilage, it is necessary to precisely regulate the printing process or cross-link with other biomaterials. This ensures that the mechanical and structural characteristics of the printed structures are finely tuned, and their biological properties and functions are closer to those of natural articular cartilage. In addition, drugs and bioactive substances such as growth factors, cytokines, or small molecule compounds can also be incorporated into the 3D-printed bioscaffolds to further improve the repair and regeneration of articular cartilage.

Three-dimensional bioprinting technology has emerged with unique advantages for printing biocompatible materials, cells, and other compounds in 3D constructs for cartilage repair or regeneration. Up to now, 3D bioprinting technologies have been able to generate 3D scaffolds composed of biocompatible polymer materials and a chondrocyte matrix, producing ideally shaped cartilage scaffolds with complex shapes and structures based on each individual’s anatomy. Therefore, 3D-bioprinted cartilage holds great significance in the research and application for cartilage defect repair and regeneration.

Three-dimensional bioprinting has the potential to produce bioprinted constructs capable of cell growth, proliferation, and differentiation, along with controllable mechanical and physical properties. However, in the case of 3D bioprinting of cartilage tissue, the technical challenges include bioink formulation, chondrocyte fate control, and difficulty in mimicking mechanical properties. Most of the bioinks for 3D bioprinting are limited to a narrow range of biomaterials. Furthermore, for clinical applications of 3D bioprinting technology, 3D-bioprinted constructs should more closely recapitulate the native ECM architecture, biological functions, mechanical strength, surface contour, geometry, and morphology of the native cartilage. Due to the limited capacity of proliferation and long-term maintenance of chondrocytes, MSCs or chondroprogenitor cells are considered as promising candidates for 3D bioprinting for cartilage tissue regeneration. This approach facilitates cartilage tissue growth and repair by increasing cell viability and proliferation, as well as cartilage phenotype maintenance and organization. It is important to note that the source of stem cells and the control of chondrogenic cell quality becomes a key part of cartilage tissue repair or regeneration after transplantation into the region of cartilage damage.

Another issue that needs to be faced is the hypertrophy of chondrocytes or tissue fibrosis during cartilage tissue repair. In order to prevent the hypertrophy or fibrosis of the chondrocytes produced by stem cell differentiation and to provide a suitable platform for integration between the implant and the surrounding tissues, appropriate biomaterials, procedures, and microenvironmental cues must be adopted. Despite these challenges, 3D bioprinting is still in the early stages of development in terms of clinical, economic, and ethical aspects, offering the promise to achieve rapid, long-term reconstruction of cartilage tissue defects. Further investigations of 3D bioprinted constructs are key steps in order to determine the biological bioinks and 3D bioprinting parameters to achieve 3D bioprinted constructs capable of promoting cartilage tissue regeneration.

Three-dimensional bioprinting is a promising approach and is considered as a vital tool in the repair or regeneration of cartilage tissue, thus holding the promise to improve the quality of life for patients with cartilage damage or diseases. Collaborations among biologists, bioengineers, and doctors will provide wide-spread prospects for the application of 3D bioprinting in cartilage tissue regeneration. Bioprinting technology, with its unique advantages, can be effectively used to deliver chondrogenic cells and growth factors within biomaterial-based scaffolds in precise and desired 3D structures. The ultimate goal of tissue engineering is the direct fabrication of a complete organ or tissue using 3D bioprinting technology, which could be directly transplanted into the human body. The application of artificial intelligence and machine learning in the 3D bioprinting technology has the potential to accelerate the development of this field, leading to the more rapid entry of 3D-bioprinted constructs into the global market. Overall, 3D bioprinting is expected to become a vital and efficient method in the repair or regeneration of cartilage tissue, thus improving the quality of life for patients with cartilage defects or degeneration.

## Figures and Tables

**Table 1:** Overview of some used natural hydrogels for cartilage tissue engineering.

Name	Chemical	Advantages	Limitations	References
Alginate	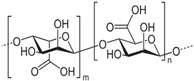	High biocompatibility Good printability Ease of preparation and gelation	Limited mechanical properties Low cell adhesion Short term structure maintenance	[72,73,74,75,76,77,78,79,80,81,82,83,84,85,86,87,88,89,90,91,92]
Hyaluronic acid (HA)	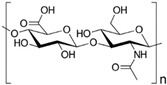	Excellent biocompatibility Good biodegradation Similar in structure to the native extracellular matrix (ECM)	Limited mechanical properties Pure HA lacks sufficient printability and may need to be modified with other biopolymers	[93,94,95,96,97,98,99,100,101,102,103,104,105]
Collagen (COL)	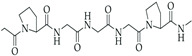	Excellent biocompatibility Supporting cell migration Facilitating the differentiation of MSCs into chondrocytes	Low mechanical properties Limited sterilizability Potential of immunogenicity High cost	[106,107,108,109,110,111,112,113,114,115,116,117,118,119,120,121,122]
Silk fibroin (SF)	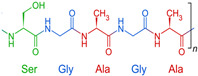	Minimal immune response High mechanical strength Suitable degradation rate Optimized structural integrity	Source variability Low biodegradability of the β-sheet crystals Need to conjugate with other materials to enhance the functionality	[123,124,125,126,127,128,129,130,131,132,133,134,135,136,137,138,139,140,141]

**Table 2:** Overview of some used synthetic hydrogels for cartilage tissue engineering.

Name	Chemical	Advantages	Limitations	References
Polyethylene glycol (PEG)	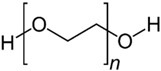	Excellent biocompatibility, reducing the risk of adverse reactions Versatile in forming hydrogels suitable for cell encapsulation. Can be modified to mimic the extracellular matrix (ECM) environment.	Susceptible to rapid degradation, limiting long-term tissue regeneration. Surface modifications are required to enhance cell attachment due to the restricted availability of cell adhesion sites.	[144,145,146,147,148,149,150,151,152,153]
Gelatin methacryloyl (GelMA)	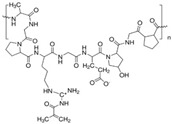	Derived from natural sources, promoting cell adhesion and proliferation. Biodegradable, allowing for gradual tissue integration and remodeling. Tunable mechanical properties through modification.	Poor mechanical properties compared to synthetic polymers. Limited stability under physiological conditions, potentially affecting long-term functionality.	[154,155,156,157,158,159,160,161,162]
Polylactic acid (PLA)	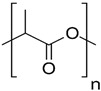	Biodegradable and biocompatible, facilitating tissue integration. High strength and stiffness, suitable for load-bearing applications. Can be fabricated into porous scaffolds for cell infiltration.	Slow degradation rate may not match tissue regeneration kinetics. Limited cell adhesion sites, necessitating surface modifications for enhanced cell attachment.	[163,164,165,166,167,168,169,170,171]
Polyvinyl alcohol (PVA)	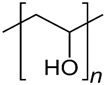	Biocompatible and non-toxic, ensuring minimal host response. Good mechanical strength, offering support for tissue growth. Forms transparent and flexible scaffolds for visualization and handling.	Susceptible to hydrolysis, potentially compromising scaffold integrity over time. Limited capacity to promote cell adhesion and proliferation without modifications.	[172,173,174,175,176,177,178,179,180]
Hyaluronic acid methacrylate (HAMA)	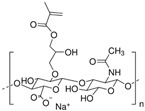	Naturally occurring in the body, reducing immunogenicity. High water retention capacity, maintaining a hydrated microenvironment. Supports cell proliferation and differentiation through interaction with cell surface receptors.	Limited mechanical strength, requiring reinforcement for load-bearing applications. Rapid degradation may not align with tissue regeneration timelines.	[181,182,183,184,185,186,187,188,189,190,191,192]
Methylcellulose (MC)	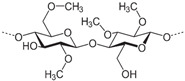	Biocompatible and non-toxic, suitable for various tissue engineering applications. Forms reversible gels, allowing for dynamic changes in scaffold properties. Transparent and flexible, facilitating visualization and manipulation.	Relatively weak mechanical properties, necessitating reinforcement for structural support. Limited stability in physiological conditions, potentially affecting long-term functionality.	[193,194,195,196,197,198,199,200,201]
Polyurethane (PU)	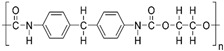	Diverse mechanical properties, allowing for customization to match tissue requirements. Excellent wear and tear resistance, suitable for load-bearing tissues. Can be fabricated into porous scaffolds with controlled pore sizes for cell infiltration.	Potential for allergic reactions in some individuals, impacting biocompatibility. Limited cell adhesion sites, requiring bioactive modification for better cell adhesion.	[202,203,204,205,206,207,208,209,210,211,212]
